# Prevalence and Clinical Spectrum of Liver Disease in Nepalese HIV-Sero-Positive Patients Undergoing Antiretroviral Therapy: A Cross-Sectional Hospital Based Study

**DOI:** 10.1155/2017/3134790

**Published:** 2017-06-11

**Authors:** Roshan Kumar Jha, Shiv Kumar Sah

**Affiliations:** ^1^Nepal Armed Police Force Hospital, Balambu, Kathmandu, Nepal; ^2^National Academy of Medical Science, Bir Hospital, Mahaboudha, Kathmandu, Nepal; ^3^Faculty of Pharmaceutical Science, Purbanchal University, Little Buddha College of Health Science, Minbhawan, Kathmandu, Nepal

## Abstract

**Introduction:**

Liver enzyme abnormalities are common in HIV patients, and the prevalence varies across the nations. In Nepal, however, prevalence of liver enzyme disorder and the spectrum of these populations are lacking.

**Objective:**

The present study sheds light on prevalence and clinical spectrum of liver disease in Nepalese HIV-sero-positive patients.

**Methods:**

This cross-sectional study was conducted at OPD/ART, Clinic of Bir Hospital, NAMS. One hundred and forty-four HIV positive patients were enrolled consecutively and their clinical profiles of liver injury were investigated.

**Results:**

Of 144 recruited patients, liver enzyme injury was observed in 82 (56.9%). Majority 61 (42.4%) of these cases had hepatocellular type of liver injury. Opportunistic infections were reported in 18 cases, with 9 (6.2%) TB and 8 (5.6%) HCV. Test for significance of liver injury confirmed the absence of any tendency towards an association with coinfection, CD4 cells, ART regimen, and alcohol consumption (*P* > 0.05). However, gender significantly linked with liver injury as well as the pattern of liver injury (*P* < 0.05).

**Conclusion:**

The study revealed high rate of liver injury in a substantial proportion of HIV individuals, stressing that a regular clinic follow-up is necessary for the HIV individuals who are undergoing ART.

## 1. Introduction

Liver enzyme abnormalities are common in HIV patients and have been reported in 20–93% of HIV-infected populations [1, 2]. The common causes encompass opportunistic infections, malignancies, and drug toxicities. Incidence of cirrhosis and mortality attributable to liver disease are considerably increased in HBV and hepatitis C virus infected HIV patients [3–6].

Several antiretroviral drugs from PI, NRTIs, and NNRTIs groups are identified for liver serum enzyme (alanine aminotransferase or aspartate aminotransferase) elevation [7–9]. However, in many circumstances, liver injury patients are underreported because as much as 50% of patients are not manifesting symptoms despite the liver enzyme elevation [10]. After initiating ART, the reported incidence of severe liver toxicity ranges from 2 to 18% [11–13]. Likewise, Protease inhibitor (PI) manifested more antiretroviral therapy related liver injury and the rates of hepatotoxicity from various PI have ranged from 1% to 9.5%, with few patients exhibiting serious liver-related outcome [14]. Besides the antiretroviral drugs, other frequently prescribed drugs for the management of opportunistic infection including antitubercular drugs are also important cause of liver injury in developing country [15, 16]. Additionally, alcohol use with concurrent hepatotoxic drugs is another independent factor for liver toxicity [11]. The pattern of liver disease etiology described in the published literature may differ from our Nepalese setting owing to availability of recent ART and individual host genetics and susceptibility of the drugs and disease. Thus, the present study aims to illustrate the frequency and the factors associated with liver injury, especially focusing on drug-related liver injury among HIV-infected individuals attending ART Clinic at Bir hospital Kathmandu.

## 2. Methods

### 2.1. Study Design

This was a cross-sectional, observational study conducted from Oct 2014 to November 2015.

### 2.2. Setting

This study was conducted at OPD/ART–Clinic of Bir Hospital, Mahaboudha, Kathmandu, Nepal. The hospital is a 465-bed specialized institution affiliated with National Academy of Medical Sciences (NAMS).

### 2.3. Study Population and Selection

A total 144 HIV-sero-positive patients that satisfied the inclusion criteria were recruited in the study. The enrolled patients further underwent structured health history and physical examination. Patient's clinical and laboratory parameters were investigated. Subject having normal baseline liver enzymes before starting of ART and the patients receiving ART for at least three months before recruitment of the study were included for analysis. All patients had undergone determination of CD4 counts at the time of enrollment in the study.

### 2.4. Liver Enzyme and Interpretation

All patients had liver function biomarkers including alanine aminotransferase (ALT), alkaline phosphatase (ALP), hepatitis B surface antigen (HBsAg), and anti-hepatitis C antibody taken. Patterns of liver injury were determined according to the liver enzyme results based on the upper limit of normal (ULN), following American College of Gastroenterology Clinical guidelines 2015. The upper limit of normal > 30 IU/L was set as a reference level.

Hepatocellular (ALT ≥ 3 × ULN; *R* ≥ 5), cholestatic (ALP ≥ 2 × ULN; *R* ≤ 2), mixed (ALT ≥ 3 × ULN, ALP ≥ 2 × ULN; *R* > 2 to <5) where *R* value = (ALT/ULN)/(ALP/ULN).

*R* value was used to define hepatotoxicity injury pattern: hepatocellular (*R* > 5), mixed (*R* > 2 to <5), and cholestatic (<2).

### 2.5. Statistical Analysis

Distribution of continuous variable such as age was expressed as median. Frequency table was generated for sex, marital status, literacy, employment, OI, and ART regimen. Test for the significance of association for categorical measure was performed by Pearson's Chi-square (*χ*^2^). A *P* value < 0.05 was set as the level of significance. All analysis was performed by using SPSS (version 16).

### 2.6. Ethical Consideration

The study protocol was approved by the institutional review board (IRB) of national Academy of Medical Science (NAMS), Bir Hospital. Patients were informed regarding the objectives and nature of the study, and the subjects that showed willingness to participate voluntarily in the study consented prior to commencing the study.

## 3. Results

Demographic of the enrolled subjects is shown in Table 1. Of 144 recruited patients, 80 (55.6%) were male, whereas 3 (2.1%) were transgender. Median ages of the subjects were 34 years. Majority 20 (72.2%) of the population were married. In this study, literate population was substantially high, 130 (90%). Opportunistic infections were recorded in 18 (12.55%) cases, 9 with TB, 8 with HCV, and 1 with both HCV and HBV (Table 1). More than half 82 (56.9%) of the population took EFV based regimen, followed by NVP 51 (35.4%).

Table 2 depicts the prevalence and pattern of liver injury to the enrolled subjects. Investigation showed that prevalence of liver injury was 82 (56.9%), at 95% CI: lower limit 52%, upper limit 60%. Among these patients 61 (42.4%) had hepatocellular type, 16 (11.1%) had cholestatic type, and another 6 (4.2%) had mixed type of liver injury.

Table 3 shows the patients characteristics and liver injury of the enrolled HIV patients. Of 144 subjects, liver injury was observed in 55 (38.19%) males, 26 (18.05%) females, and 1 (0.69%) transgender. Most of the patients had CD4 cell count between 200 and 500, and the proportion of liver injury observed in this group was 21 (14.58%). Liver injury in coinfected subjects recorded was TB: 8 (5.55%), HCV 5 (3.47%), and HCV + HBV 1 (0.69%). Most patients received EFV based regimen, and the proportion of liver injury was observed in 51 (35.41%) of the total population, followed by 25 (17.36%). Liver injury, expressed as normal and abnormal, was significantly associated with gender (*P* = 0.06).

As seen in the Table 4, different types of liver injury against the gender were hepatocellular type, 43 (29.86%) males versus 17 (11.80%) females, cholestatic liver toxicity, 10 (6.94%) males versus 6 (4.16%) females, and mixed type, 4 (2.77%) males versus 2 (1.38%) females. Hepatocellular type of liver injury was predominant in patients having CD4 range of 50–500. Coinfections were recorded in 18 subjects. Of these, 9 TB patients had hepatocellular type of liver injury. Hepatitis C was seen in 8 (5.6%) subjects, 4 with hepatocellular type and 1 with cholestatic type of liver injury. Hepatocellular type of liver injury was seen in a substantial number 41 (28.47%) of population receiving EFV based regimens. Statistics revealed a significant association between gender and the pattern of liver injury (*P* < 0.05).

## 4. Discussion

Liver enzyme abnormalities have been reported in 20–93% of HIV-infected populations [1, 2]. Data from resources reach setting countries demonstrated that about 10% of patients who started antiretroviral drug developed significant liver injury, with risk factors being hepatitis C infection, older age group, and alcohol [11]. In agreement with the previous documented literature, we observed a significant 82 (56.9%) elevation of liver enzyme in our study population.

In a previous study explored by Ocama et al. [17], mean ages were 37 years, where 47% were male patients. In this study, median population ages were 34 years, and the prevalence of HIV observed was higher, 55 (38.19%), in male than in female, 26 (18.05%). In our study population, it is possible that the male predominance could be due to the factors that include drug user, migrant worker from India, and alcohol consumption.

Majority 105 (72.29%) of the patients ages group were between 18 to 39 years. This finding complies with the previous study done in Nepal [18]. Our investigation revealed that married 24 (72.2%), literate 130 (90.3%), and employed people 102 (70.8%) were mostly infected with HIV compared to the unmarried, illiterate, and unemployed people. These results showed that most of the working aged male population was not practicing healthy sexual behavior despite being educated. Higher proportions of disease in married population in our study suggest that the couple may not be aware of the transmission of the disease, resulting in higher prevalence.

In concordance with the study conducted by Ocama et al. [19], our study showed that hepatotoxicity was seen more in male than in female, 55 (54.99%) versus 26 (18.05%). Pattern of different type of liver injury was recorded higher in males than in females. One of the reasons of this higher percentage of different type liver injury among male subjects may be due to presence of coinfections such as Hep B/C, alcohol intake, and other factors including intravenous drug use. HIV-infected individuals in the resource-rich setting nations are more prone to chronic HCV infection, which itself is associated with grade III-IV liver enzyme elevation [20]. But in this study we observed a low prevalence 8 (5.5%) of HCV coinfection. Available literature also suggests a similar low HCV prevalence in many resource-low setting nations [17, 21–23]. Among the cases that had hepatitis C coinfection, 4 cases had hepatocellular type and 1 had mixed type of liver injury. Coinfections with HBV occur in 3–15% in HIV-infected persons in developing countries [22, 23]. However, in our study HBV coinfection was uncommon which was seen in 1 (0.6%).

In this study, hepatocellular type of liver injury was substantially high 61 (42.36%) followed by cholestatic 16 (11.11%). Six (4.16%) subjects had mixed type of liver injury. In some parts of world, hepatocellular carcinoma is common, and HBV and other carcinogenic agents such as alcohol and aflatoxin could impart this [24, 25]. However, liver injury pattern observed in a study was 63% cholestatic, 8% cellular, and 19% mixed [26]. These wide varied patterns maybe due the characteristic of the patients enrolled in the study. However, further studies in a large population are needed to explore the pattern of liver injury and its contributing factors.

Registration trial of nevirapine and efavirenz shows an acceptable toxicity profile. Two distinct patterns of drug injury associated with nevirapine use have emerged: hypersensitivity reactions and direct drug-related toxicity [27]. In our study, liver toxicity was mostly observed in efavirenz based regimen compared to in nevirapine, 51 (35.41%) versus 25 (17.36%), and hepatocellular type of injury was predominantly 41 (28.47%) observed in EFV based regimen.

## 5. Limitations

Our study has several limitations. A true liver function test such as INR, albumin, was not performed and was not taken into account for the evaluation. Presence of liver fibrosis and ascites were not evaluated as there was no result of platelets count and ultrasounds. We believe that the test is expensive and considering the economic burden of the people for resource limited country like Nepal, it is not possible to perform all tests.

## 6. Conclusion

Liver injury was observed in a substantial number of HIV-sero-positive patients, leaving an unavoidable issue to be resolved. Of various pattern of injury, hepatocellular type remained the most common. The finding indicates that HIV-sero-positive patients undergoing ART are at high risk of liver injury, and there is a need for a regular clinical follow-up.

## Figures and Tables

**Table 1:** Demographics and patient characteristics.

Age (years)	Number (%)
*N*	144
*Age, median, range*	34, 18–61
*Age (category)*	
18–39	105 (72.29)
40–60	38 (26.38)
>60	1 (0.69)
*Sex*	
Male	80 (55.6)
Female	61 (42.4)
Transgender	3 (2.1)
*Marital status*	
Married	24 (16.7)
Unmarried	104 (72.2)
Widow	14 (9.2)
Divorced	2 (1.4)
*Literacy*	
Literate	130 (90.3)
Illiterate	14 (9.7)
*Employment*	
Employed	102 (70.8)
Unemployed	42 (29.2)
*Opportunistic infection*	
TB	9 (6.2)
HCV	8 (5.6)
HCV + HBV	1 (0.7)
*ART*	
NVP	51 (35.4)
EFV	82 (56.9)
Others	11 (7.6)

**Table 2:** Liver enzyme status.

Liver enzyme	Patients: 144	95% CI	
	*N* (%)	Lower bond	Upper bond
*Abnormal*	82 (56.9)	52%	60%
*Pattern of liver injury*			
Hepatocellular	61 (42.4)		
Cholestatic	16 (11.1)		
Mixed	6 (4.2)		

**Table 3:** Patients characteristics and frequency of liver injury.

	Liver injury		Total	*P*(*χ*^2^)
	Abnormal	Normal	Patients	
	*N* (%)	*N* (%)	*N* = 144	
*Sex*				0.006
Male	55 (38.19)	25 (17.36)	80	
Female	26 (18.05)	35 (24.30)	61	
Transgender	1 (0.69)	2 (1.38)	3	
*Alcoholics*				0.403
Yes	32 (22.22)	20 (13.88)	52	
No	50 (34.72)	42 (29.16)	92	
*CD4 cell count*				0.074
<50	5 (3.47)	2 (1.38)	7	
50–100	2 (1.38)	8 (5.55)	10	
>100–<200	21 (14.58)	12 (8.33)	33	
200–500	49 (34.02)	39 (27.08)	88	
>500	5 (3.47)	1 (0.69)	6	
*Opportunistic infection*				0.869
TB	8 (5.55)	1 (2.70)	9	
HCV	5 (3.47)	3 (4.10)	8	
HCV + HBV	1 (0.69)	0 (0.00)	1	
None	68 (47.22)	58 (40.27)	126	
*ART*				0.324
NVP	25 (17.36)	26 (18.05)	51	
EFV	51 (35.41)	31 (37.50)	82	
Others	6 (4.16)	5 (3.47)	11	

**Table 4:** Patients characteristics and pattern of liver injury.

	Pattern of liver toxicity					*P*(*χ*^2^)
Gender	Hepatocellular	Cholestatic	Mixed	Normal	Total patients	
					*N* = 144	
*Gender*						*0.026*
Male	43 (29.86)	10 (6.94)	4 (2.77)	23 (15.97)	80	
Female	17 (11.80)	6 (4.16)	2 (1.38)	36 (25.00)	61	
Transgender	1 (0.69)	0 (0.00)	0 (0.00)	2 (1.38)	3	
*CD4 cells*						*0.382*
<50	4 (2.77)	1 (0.69)	0 (0.00)	2 (1.38)	7	
>50–<100	2 (1.38)	0 (0.00)	0 (0.00)	8 (5.55)	10	
100–200	17 (35.71)	3 (1.30)	1 (0.60)	12 (13.88)	33	
>200–500	35 (11.88)	10 (6.94)	5 (3.47)	38 (26.38)	88	
>500	3 (2.08)	2 (1.38)	0 (0.00)	1 (0.69)	6	
*Coinfection*						*0.18*
HCV	4 (2.77)	1 (0.69)	0 (0.00)	3 (2.08)	8	
HCV + HBV	1 (0.69)	0 (0.00)	0 (0.00)	0 (0.60)	1	
TB	8 (5.55)	0 (0.00)	0 (0.00)	1 (0.69)	9	
None	48 (33.33)	15 (10.41)	6 (4.16)	57 (39.58)	126	
*Alcoholics*						*0.068*
Yes	29 (20.13)	3 (2.08)	3 (2.08)	18 (12.50)	53	
No	32 (2.08)	13 (9.08)	3 (2.08)	43 (29.06)	91	
*ART*						*0.129*
NVP	17 (11.80)	6 (4.16)	1 (0.69)	27 (18.75)	51	
EFV	41 (28.47)	7 (4.86)	5 (3.47)	29 (2013)	82	
Others	3 (2.08)	3 (2.08)	0 (0.00)	5 (3.47)	11	

## Abbreviations

EFV: Efavirenz
NVP: Nevirapine
ART: Antiretroviral therapy
HBV: Hepatitis B virus
HCV: Hepatitis C virus
PI: Protease inhibitor
NRTI: Nucleoside reverse transcriptase inhibitor
NNRTI: Nonnucleoside reverse transcriptase inhibitor.
